# Combination treatment with anti-RANKL and antibiotics for preventing joint destruction in septic arthritis

**DOI:** 10.1172/jci.insight.184954

**Published:** 2025-03-24

**Authors:** Zhicheng Hu, Meghshree Deshmukh, Anders Jarneborn, Miriam Bollmann, Carmen Corciulo, Pradeep Kumar Kopparapu, Abukar Ali, Mattias N. D. Svensson, Cecilia Engdahl, Rille Pullerits, Majd Mohammad, Tao Jin

**Affiliations:** 1 Department of Rheumatology and Inflammation Research, Institute of Medicine, The Sahlgrenska Academy, University of Gothenburg, Gothenburg, Sweden.; 2 Center for Clinical Laboratories, the Affiliated Hospital of Guizhou Medical University, Guiyang, China.; 3 Department of Rheumatology, Sahlgrenska University Hospital, Gothenburg, Sweden.; 4 SciLifeLab and; 5 Department of Pharmacology, Institute of Neuroscience and Physiology, University of Gothenburg, Sweden.; 6 Department of Clinical Immunology and Transfusion Medicine, Sahlgrenska University Hospital, Gothenburg, Sweden.

**Keywords:** Bone biology, Infectious disease, Arthritis, Bone disease, Mouse models

## Abstract

Septic arthritis, the most severe joint disease, is frequently caused by *Staphylococcus aureus* (*S*. *aureus*). A substantial proportion of patients with septic arthritis experience poor joint outcomes, often necessitating joint replacement surgery. Here, we show that monocyte depletion confers full protection against bone erosion in a septic arthritis mouse model. In the infected synovium, Ly6C^hi^ monocytes exhibited increased expression of osteoclastogenesis-related molecules, including CCR2, c-Fms, and RANK. *S*. *aureus* lipoproteins induced elevated levels of RANKL, MCSF, and CCL2 in joints, with synovial fibroblasts identified as the major RANKL producer. Anti-RANKL treatment prevented bone destruction in both local and hematogenous septic arthritis murine models. Importantly, combining anti-RANKL treatment with antibiotics provided robust protection against joint damage. Our results indicate that the infiltration and transformation of monocytes into bone-destructive, osteoclast-like cells are key mechanisms in septic arthritis. Combining anti-RANKL and antibiotic therapy represents a promising therapy against this devastating disease.

## Abstract 2

A novel therapy to prevent bone damage in septic arthritis: anti-RANKL treatment, offering a promising strategy for enhancing joint health alongside antibiotic treatment.

## Graphical abstract

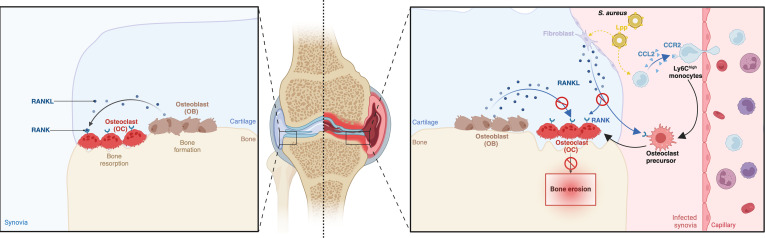

## Introduction

Septic arthritis, the most aggressive form of joint disease, is predominantly caused by *Staphylococcus aureus* (*S*. *aureus*), with an annual incidence of 4–10 cases per 100,000 inhabitants in developed countries (1–3). Despite advancements in antibiotic therapies, a substantial proportion of patients with septic arthritis experience poor joint outcomes (4). Secondary osteoarthritis arises in around 30% of individuals who have had septic arthritis in the shoulder or knee (5). According to a comprehensive epidemiologic study, approximately 9% of patients previously affected by septic arthritis underwent arthroplasty within 15 years, indicating a risk that is 6-fold higher than that of the general population (6).

Treatments for septic arthritis typically involve antibiotics and joint drainage (1). Unfortunately, there has been very little progress in the development of treatment alternatives over the past 30 years. To mitigate the exaggerated immune response and minimize joint damage, strategies such as combining antibiotics with immunomodulatory therapies, e.g., corticosteroids (7) or anti-TNF treatment (8), have been suggested. However, these combination therapies carry risks, particularly from concerns about immunosuppression and the distinct challenge of antibiotic resistance (9). Immunosuppressive treatments can impair the immune system’s ability to eliminate bacteria, potentially exacerbating infections. For instance, in cases of methicillin-resistant *S*. *aureus*, with the use of inappropriate antibiotics alongside these therapies, the infection could worsen further. To date, these approaches have not been implemented in clinical practice.

Articular bone erosions play a substantial role in the subsequent loss of joint function in autoimmune arthritis, notably rheumatoid arthritis (RA) (10). In contrast to RA, where bone erosion develops over months to years, severe bone erosion in affected joints is observed within several days after the onset of septic arthritis in a hematogenous septic arthritis model (11), reflecting its rapid and intense inflammatory nature. Several lines of evidence indicate that bacterial components are responsible for the bone destruction seen in septic arthritis. For instance, intra-articular injection of bacterial DNA containing CpG motifs induces erosive arthritis in mice (12). Moreover, antibiotic-killed *S*. *aureus* triggers destructive arthritis via TNF receptor 1, with bacterial cell wall components being identified as the culprits (13). Recent studies have shown that a single injection of *S*. *aureus* lipoproteins (Lpps) induces macroscopic erosive arthritis in mice through the lipid moiety of the Lpps (14). Importantly, these joint-eroding effects of bacterial components are mediated exclusively by monocytes, as monocyte depletion abolishes both joint inflammation and bone destruction (12–15).

In autoimmune arthritis, focal bone destruction is partly due to excessive bone resorption through osteoclast activation, which is mediated by increased local expression of receptor activator of NF-κB ligand (RANKL) (16). Notably, inhibiting RANKL with anti-RANKL antibodies impedes the advancement of joint destruction in RA (17). It has been shown that osteoclasts can originate not only from within the bone but also from mature monocytes and macrophages under the appropriate microenvironmental conditions (18).

In the current study, we identify monocytes as the central players in bone destruction in murine models of septic arthritis. Further investigation reveals that monocyte differentiation into bone-destructive, osteoclast-like cells is the primary mechanism driving rapid joint damage. Most importantly, combination therapy with antibiotics and anti-RANKL treatment is superior to antibiotic therapy alone in protecting joints from bone destruction, without exacerbating the infection.

## Results

### Monocytes are pathogenic in septic arthritis–induced bone destruction.

**Figure 1:**
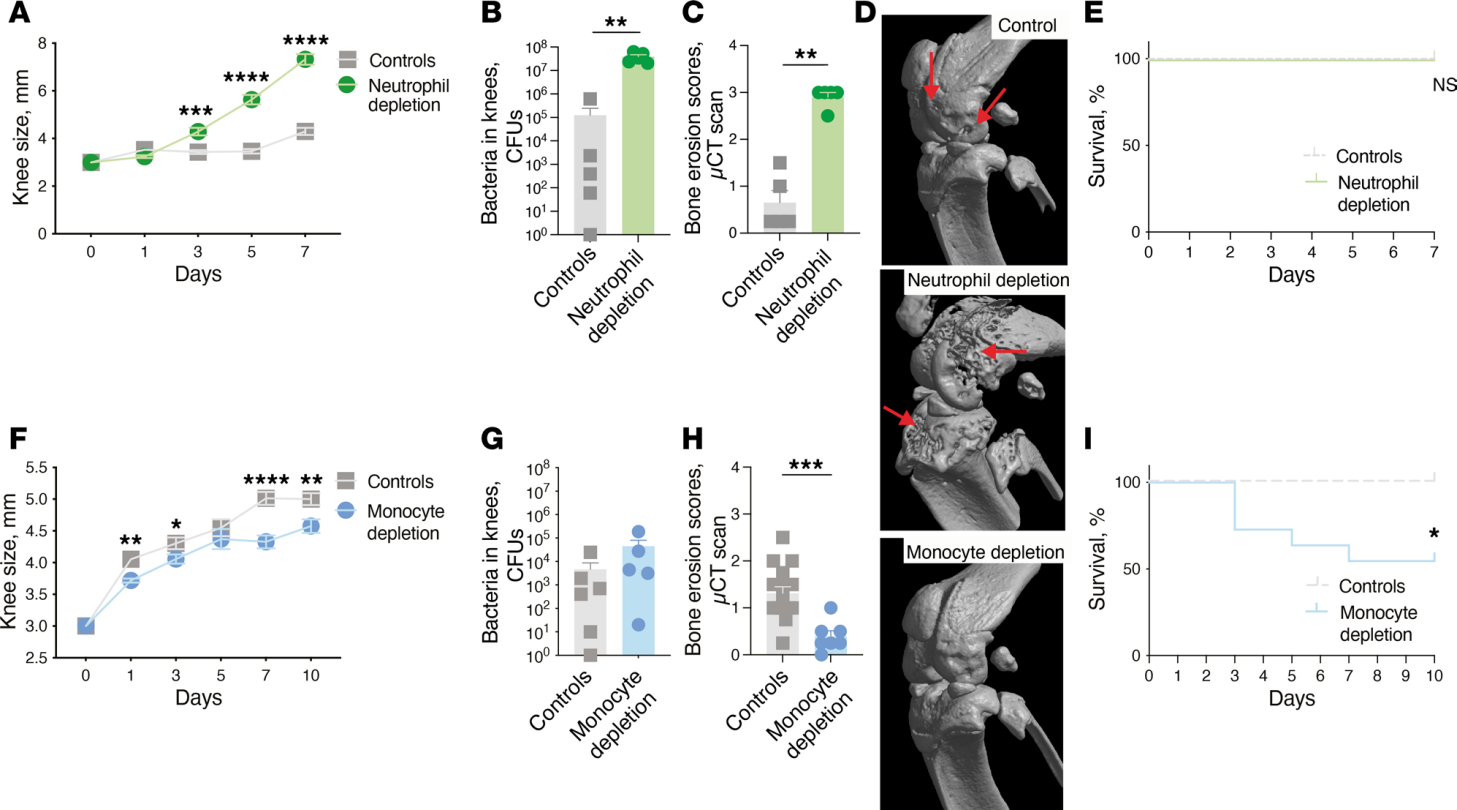
Monocytes are pathogenic while neutrophils are protective in septic arthritis–induced bone destruction. (**A**) The protective effect of neutrophils was examined by depleting mice of neutrophils (*n* = 10/group) using the anti-Ly6G antibody (Neutrophil depletion) or an isotype control (Controls). Knee swelling was measured in millimeters over a period of 7 days after intra-articular (i.a.) injection of 20 μL of PBS containing *S*. *aureus* LS-1 strain (4 × 10^3^ CFU/knee) into the knee joints of NMRI mice. (**B**) Bacterial counts in the mouse knee joints (*n* = 5/group) were assessed. (**C**) Bone erosion scores of the knee joints (*n* = 5/group) were determined after micro-CT (μCT) scan. (**E**) Cumulative survival was monitored daily. (**F**) The pathogenic role of monocytes was investigated by depleting mice of monocytes (*n* = 22/group) using clodronate liposomes (Monocyte depletion) or PBS control liposomes (Controls), and knee swelling was followed for 10 days after infection using the same strategy. (**G**) Bacterial counts in mouse knee joints in the monocyte depletion group (*n* = 5) and controls (*n* = 6). (**H**) Bone erosion scores for the monocyte depletion group (*n* = 7) and controls (*n* = 16) and (**I**) cumulative survival. (**D**) Representative μCT images of knee joints from the control, neutrophil depletion, and monocyte depletion groups. Arrows indicate bone erosion. The data were pooled from 2 independent experiments. Statistical evaluations were performed using the Mann-Whitney test (**A**–**C** and **F**–**H**) or log-rank (Mantel-Cox) test (**E** and **I**). Data are presented as mean with SEM. *P < 0.05; **P < 0.01; ***P < 0.001; ****P < 0.0001.

To determine which immune cell types drive bone destruction in septic arthritis, we intra-articularly (i.a.) injected *S*. *aureus* into mouse knee joints following the depletion of either monocytes or neutrophils. The depletion of neutrophils resulted in more severe bone destruction, and this was accompanied by higher levels of joint swelling and bacterial proliferation compared with the control mice (Figure 1, A–D). Conversely, mice that were depleted of monocytes showed reduced joint inflammation and significantly less joint damage, despite a comparable bacterial burden (Figure 1, D and F–H). This strongly indicates a pathogenic role for monocytes and a protective role for neutrophils in the development of bone destruction in septic arthritis.

Remarkably, the monocyte-depleted mice showed a higher mortality rate than the mice lacking neutrophils (Figure 1, E and I). This highlights the dual role of monocytes in septic arthritis: While contributing to the pathogenesis of bone destruction, they also play a crucial role in protecting the host from lethality.

### Osteoclastogenic cytokine receptors are upregulated in synovial Ly6C^hi^ monocytes in septic arthritis.

**Figure 2:**
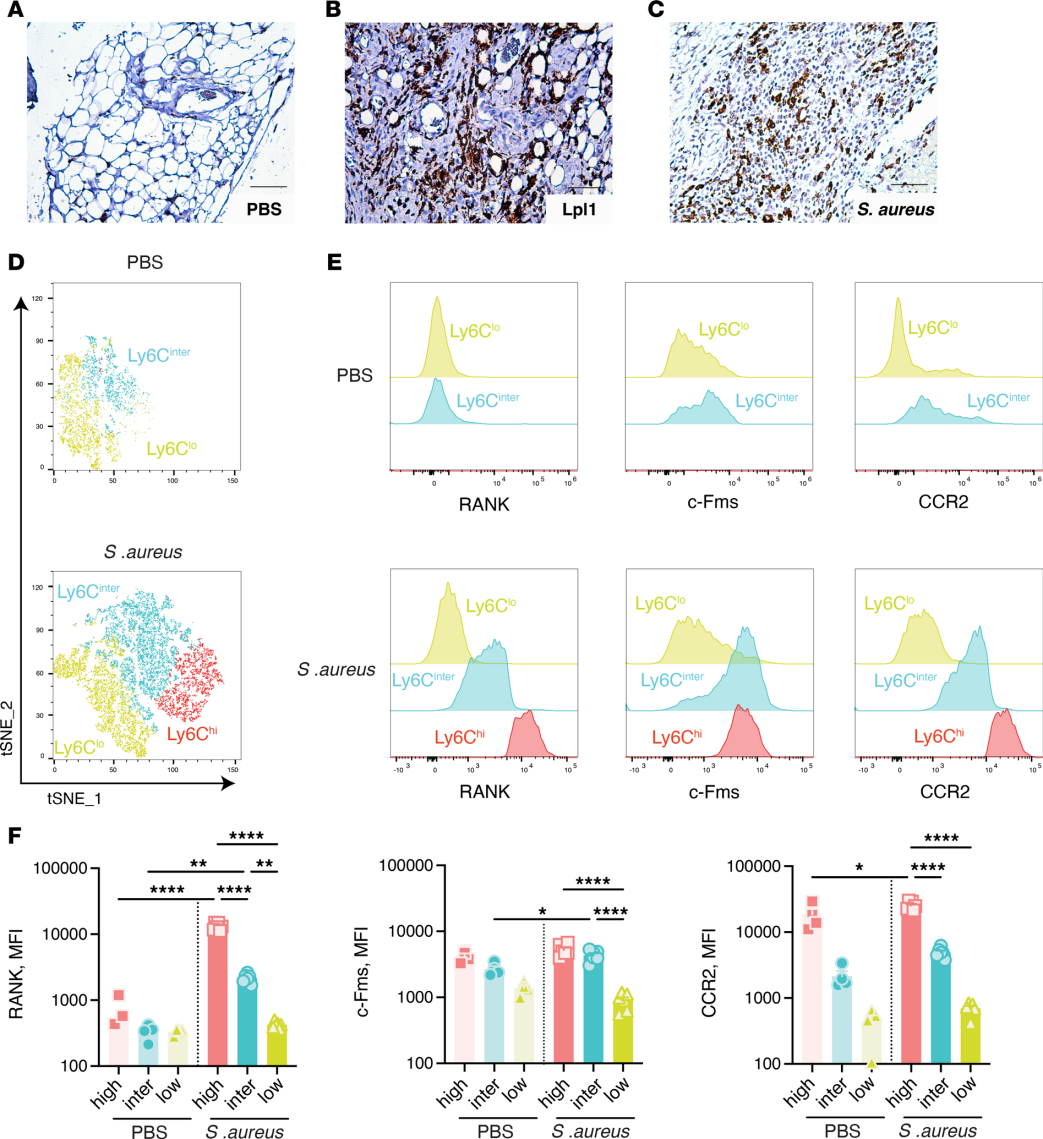
Infiltrating synovial Ly6C^hi^ monocytes exhibit increased expression of osteoclastogenesis-related cytokine receptors in septic arthritis. Knee joints from NMRI mice, collected on day 3 after intra-articular (i.a.) injection of 20 μL of PBS or Lpl1(+sp) (4 μg/knee) or PBS containing *S*. *aureus* LS-1 strain (4 × 10^3^ CFU/knee), underwent anti-CD68 immunohistochemistry examination (**A**–**C**). Scale bar: 100 μm. Lpl1(+sp), *S*. *aureus* intact Lpp. Knee synovial tissues from mice that were injected i.a. with 20 μL of PBS (*n* = 4) or PBS containing *S*. *aureus* LS-1 strain (4 × 10^3^ CFU/knee, *n* = 6) were analyzed on day 3 after injection using flow cytometry. t-Distributed stochastic neighbor embedding (tSNE) analysis of monocyte subsets based on Ly6C expression with gating on CD11b^+^CD45^+^Ly6G^–^ population was conducted (**D**). Representative mean fluorescence intensity (MFI) histograms of monocyte subsets for RANK, c-Fms, and CCR2 are shown (**E**). Statistical analyses of RANK, c-Fms, and CCR2 levels are presented (**F**). Statistical evaluations were performed using 1-way ANOVA with Holm-Šídák multiple-comparison test, with data presented as mean with SEM. *P < 0.05; **P < 0.01; ****P < 0.0001.

Immunohistochemical staining with anti-CD68 revealed a pronounced increase in monocyte infiltration of the synovial tissue 3 days after i.a. injection of either live *S*. *aureus* LS-1 strain or purified *S*. *aureus* Lpp (Figure 2, A–C), indicating the early presence of monocytes in the disease process.

To determine if these monocytes are involved in infection-induced osteoclastogenesis within the synovium, we conducted flow cytometric analyses, focusing on the expression levels of RANK, c-Fms (also known as colony-stimulating factor 1 receptor, CSF1R), and CCR2 in monocyte subgroups on day 3 after infection. Compared with PBS-injected knee joints, the joints that were infected with *S*. *aureus* showed higher numbers and proportions of Ly6C^hi^ cells within the CD11b^+^CD45^+^Ly6G^–^ monocyte population (Figure 2D), as well as increased expression levels of monocyte activation markers CD64 and CX3CR1 (Supplemental Figure 2; supplemental material available online with this article; https://doi.org/10.1172/jci.insight.184954DS1). Significantly increased mean fluorescence intensities (MFIs) of RANK, c-Fms, and CCR2 were detected in the Ly6C^hi^ monocyte subset (Figure 2, E and F), indicating that they are a major supplier of bone-destructive osteoclasts, when a suitable microenvironment prevails within the infected joints.

### Levels of osteoclastogenic cytokines are increased in arthritis induced by local injection of S. aureus Lpps.

**Figure 3:**
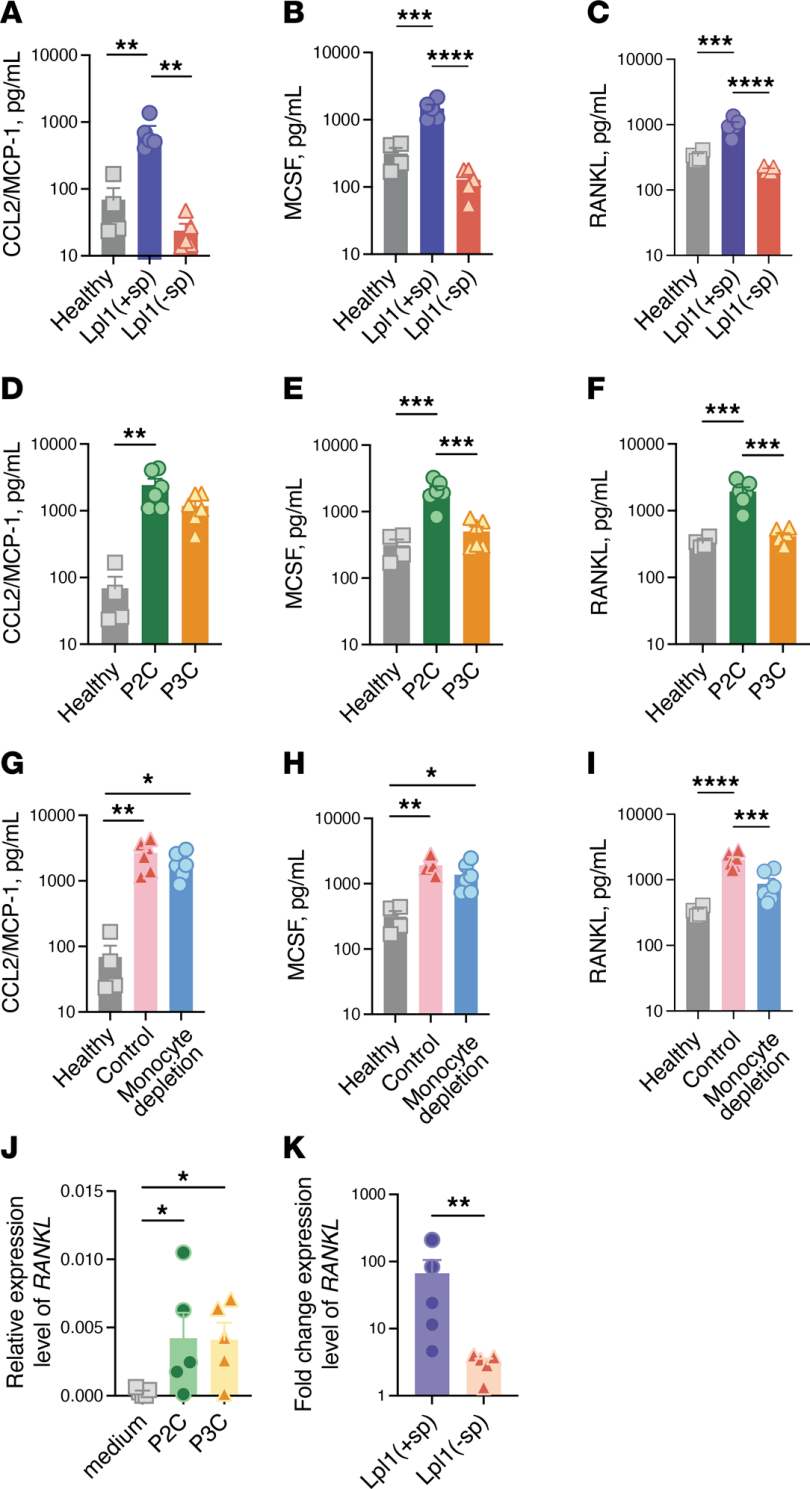
*S*. *aureus* Lpps induce the expression of osteoclastogenesis-related cytokines in injected joints. Supernatants from knee homogenates of NMRI mice on day 3 after intra-articular (i.a.) injection of 20 μL of PBS (healthy; *n* = 4) or Lpl1(+sp) or Lpl1(-sp) (4 μg/knee; *n* = 5/group) were assessed for the levels of CCL2 (MCP-1, **A**), MCSF (**B**), and RANKL (**C**) by ELISA. Levels of CCL2 (MCP-1, **D**), MCSF (**E**), and RANKL (**F**) in the supernatants from knee homogenates of NMRI mice on day 3 after i.a. injection of 20 μL of PBS (healthy; *n* = 4) or the synthetic lipopeptides, P2C or P3C (4 μg/knee; *n* = 4 to 6/group). Levels of CCL2 (MCP-1, **G**), MCSF (**H**), and RANKL (**I**) in the supernatants from knee homogenates of NMRI mice depleted of monocytes/macrophages using clodronate liposomes (Monocyte depletion) or PBS control liposomes (Control) on day 3 after i.a. injection of 20 μL of P2C (4 μg/knee; *n* = 6/group). Data were pooled from 2 independent experiments. Mouse knee synovial fibroblasts were stimulated with P2C or P3C (20 ng/mL; *n* = 5/group), or Lpl1(+sp) or Lpl1(-sp) (0.2 μg/mL; *n* = 5/group) for 48 hours. The expression levels of *RANKL* were analyzed with the TaqMan assay, and the relative gene expression was calculated using the ΔCt method (**J**). The fold-changes in gene expression levels were normalized against medium-only samples (**K**). Statistical evaluations were performed using 1-way ANOVA with Tukey’s multiple-comparison test (**A**–**I**), repeated measures 1-way ANOVA with Dunnett’s multiple-comparison test (**J**), and the Mann-Whitney test (**K**), with the data presented as mean with SEM. *P < 0.05; **P < 0.01; ***P < 0.001; ****P < 0.0001.

Our previous studies using the murine model showed that *S*. *aureus* Lpps cause marked bone destruction that is dependent upon the presence of monocytes and TLR2 (14, 19). To assess the suitability of the microenvironment for osteoclastogenesis in septic arthritis joints, mouse knee joints were i.a. injected with the Lpl1(+sp) or Lpp lacking the lipid moiety: Lpl1(-sp). The protein levels of CCL2 (monocyte chemoattractant protein–1; MCP-1), MCSF, and RANKL in the knee synovium were analyzed. Consistent with previous findings, Lpl1(+sp), but not Lpl1(-sp), induced the upregulation of osteoclastogenesis-related cytokines, i.e., CCL2 (MCP-1), MCSF, and RANKL, on day 3 after injection (Figure 3, A–C).

It has been shown that di-acylated Lpps are more potent than tri-acylated Lpps in inducing pro-inflammatory cytokines (20). By using synthetic peptides P2C and P3C (representing di- and tri-acylated Lpps), we therefore investigated the impacts of lipid moiety structures on synovial production levels of CCL2 (MCP-1), MCSF, and RANKL. Knee homogenates from P2C-injected mice exhibited increased levels of MCSF and RANKL, as compared with P3C-injected knees (Figure 3, D–F). Furthermore, monocyte depletion attenuated the expression level of P2C-induced RANKL, but not that of CCL2 or MCSF, suggesting a pivotal role for monocytes in mediating this response (Figure 3, G–I).

Synovial lining fibroblasts have been shown to be responsible for the destruction of cartilage and bone in a mouse arthritis model (21). To investigate further the roles of different cell types in the inflammatory milieu, we examined the responses of mouse synovial fibroblasts to Lpps. When stimulated with Lpl1(+sp), the fibroblasts exhibited markedly increased RANKL expression compared with those stimulated with Lpl1(-sp). Furthermore, both P2C and P3C induced the upregulation of RANKL expression in synovial fibroblasts following 48 hours of stimulation (Figure 3, J and K).

### Anti-RANKL treatment provides robust protection against bone destruction in septic arthritis in mice.

**Figure 4:**
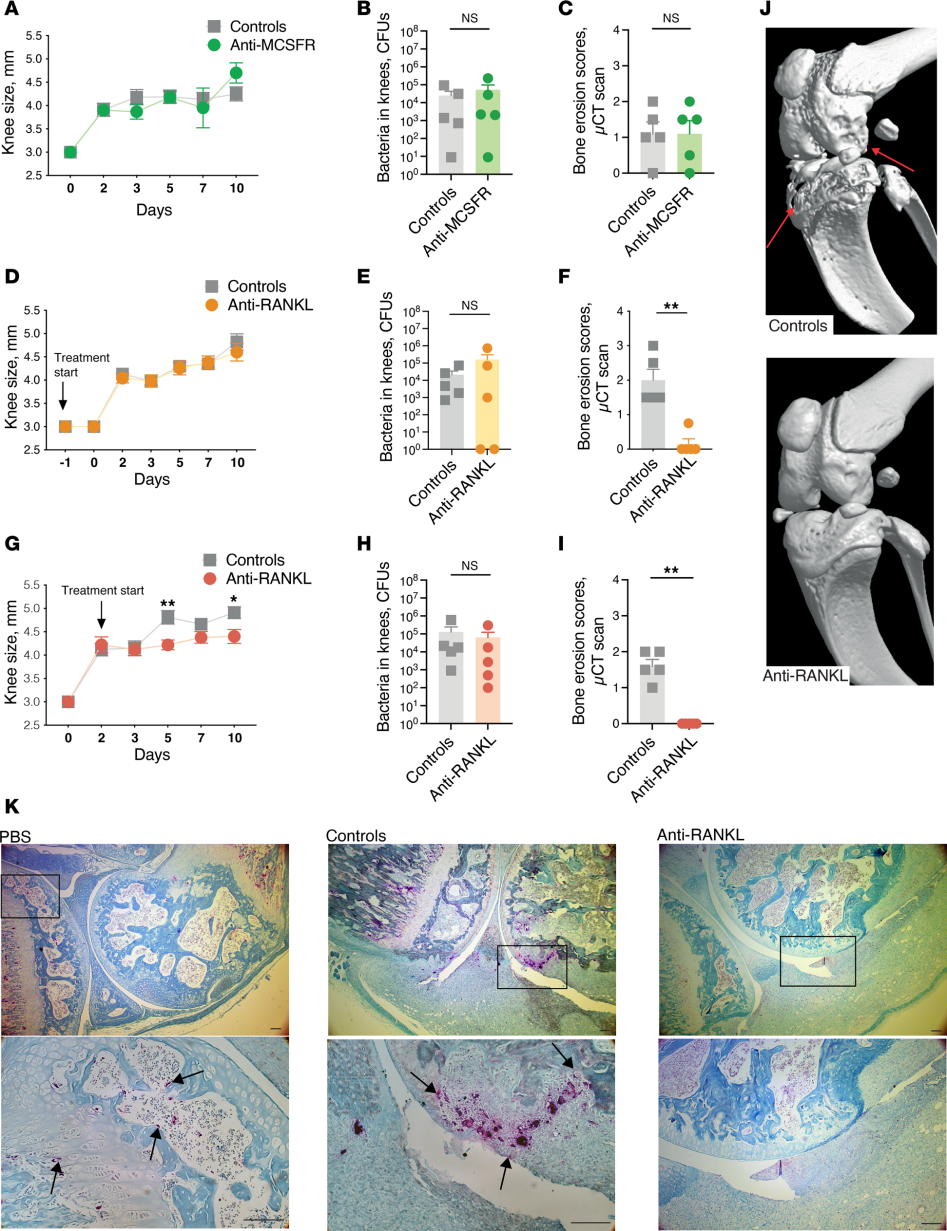
Anti-RANKL treatment provides robust protection against bone destruction in locally induced septic arthritis in mice. (**A**) Measurement of knee swelling (in mm, *n* = 10/group) of NMRI mice treated with anti-mouse CSF1R antibodies (anti-MCSFR) or an isotype control (Controls), administered 3 hours prior to intra-articular (i.a.) infection of 20 μL of PBS containing *S*. *aureus* LS-1 strain (4 × 10^3^ CFU/knee). (**B**) Bacterial counts of *S*. *aureus* in mouse knee joints (*n* = 5/group) and (**C**) bone erosion scores of mouse knee joints (*n* = 5/group) were determined after μCT scan on day 10 after the mice were sacrificed. (**D**) Knee swelling measurements (in mm, *n* = 10/group) of NMRI mice treated with anti-mouse RANKL antibodies (anti-RANKL) or an isotype control (Controls), administered 1 day prior to i.a. infection of 20 μL of PBS containing *S*. *aureus* LS-1 strain (4 × 10^3^ CFU/knee). (**E**) Bacterial counts (*n* = 5/group) and (**F**) bone erosion scores (*n* = 5/group) were determined after μCT scan on day 10 after the mice were sacrificed. To mimic more accurately the clinical progress, additional assessments were conducted with varied administration timing, 2 days after infection (**G**–**I**), and (**J**) representative μCT images are shown. Arrows indicate bone erosion. (**K**) Representative TRAP staining images of mouse knee joints that received injections of PBS, isotype control antibody, or anti-RANKL antibody. The insets (lower panels) represent higher magnification images of the boxed areas, with the arrows indicating TRAP-positive cells. Scale bar: 100 μm. Statistical evaluations were performed using the Mann-Whitney test, and the data are presented as mean with SEM. *P < 0.05; **P < 0.01.

To inhibit osteoclastogenesis at different levels, anti-MCSFR and anti-RANKL antibodies were administered to mice with local septic arthritis. Pretreatment with anti-MCSFR antibody demonstrated limited efficacy in terms of preventing arthritis development, decreasing bacterial load in joints, and providing protection against joint damage (Figure 4, A–C). We conducted a stepwise evaluation to study the effects of anti-RANKL treatment given before and after infection in mice with locally induced knee infection. Despite showing similar outcomes to the controls in relation to knee swelling and bacterial counts, there were substantial reductions in bone erosion in both scenarios (Figure 4, D–J). Importantly, none of the antibody treatments affected the weight development outcomes (Supplemental Figure 3, A–F).

Tartrate-resistant acid phosphatase (TRAP), which is highly expressed in osteoclasts, serves as an ideal histochemical marker for activated osteoclasts (22). In the knee joints infected with *S*. *aureus*, TRAP-positive cells were observed in the pannus tissue invading the cartilage and bone, indicating the differentiation of monocytes into osteoclast-like cells in the infected synovium adjacent to the bone tissue. In contrast, anti-RANKL treatment resulted in pannus tissue devoid of TRAP-positive cells (Figure 4K).

**Figure 5:**
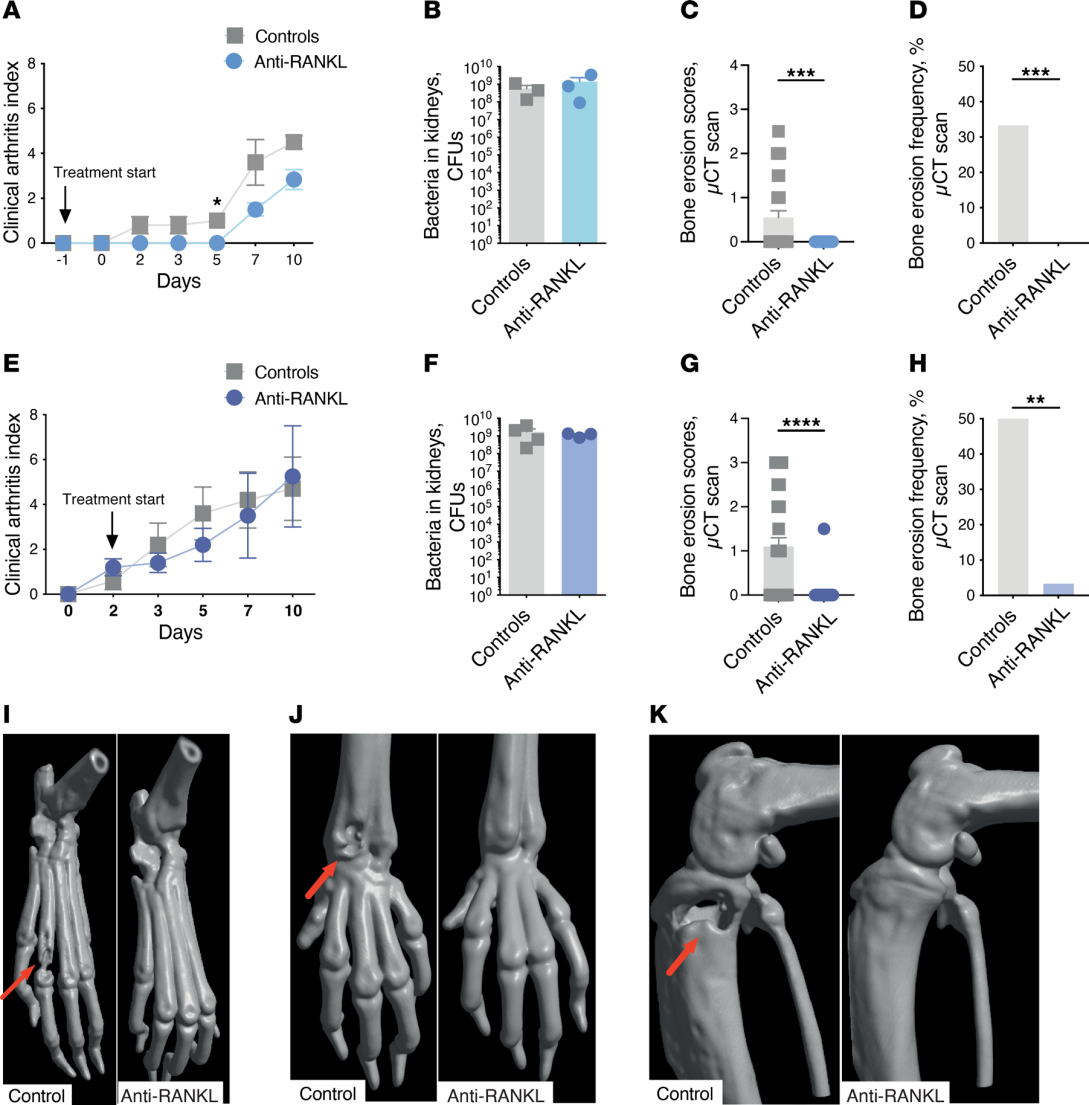
Anti-RANKL treatment prevents bone destruction in mice with hematogenous septic arthritis. NMRI mice (*n* = 5/group) received anti-RANKL treatment or the isotype control 1 day prior to intravenous infection with *S*. *aureus* Newman strain (5 × 10^6^ CFU/mouse) and were sacrificed on day 10 after infection. The arthritis severities (**A**) and bacterial loads in the kidneys (**B**). Cumulative bone destruction scores (**C**) and frequencies of bone destruction (**D**) of the joints from all 4 limbs were assessed by μCT scan. Additional assessments with administration timing, 2 days after infection (**E**–**H**), were conducted to mimic more closely the clinical progress. Representative μCT images are presented that show both control (left) and anti-RANKL–treated (right) mouse joints, (**I**) hind paws, (**J**) wrists, and (**K**) knees. Arrows indicate the bone erosion. Data are reported as mean ± SEM and analyzed with the Mann-Whitney test (**A**–**C** and **E**–**G**) or Fisher’s exact test (**D** and **H**). *P < 0.05; **P < 0.01; ***P < 0.001; ****P < 0.0001.

Following this, we used a model of hematogenous septic arthritis, which is more clinically relevant, to assess the joint-protective effects of anti-RANKL treatment. Despite similar clinical arthritis severities and bacterial loads in the kidneys, significantly reduced frequencies of bone destruction were observed in the mice treated with anti-RANKL treatments, irrespective of the time of initiation of the treatment (Figure 5, A–H). Representative μCT images highlighted the protective effects of anti-RANKL treatment in different joints affected by septic arthritis (Figure 5, I–K). While anti-RANKL treatment is crucial for preventing bone damage, it does not reduce the development of arthritis or affect the mortality rate (Supplemental Figure 3, G–J). This suggests a potential benefit from combining anti-RANKL and antibiotic treatments in the next step.

To understand whether anti-RANKL treatment affects monocyte infiltration and the expression of osteogenesis-associated cellular markers in knee synovial tissue during septic arthritis, flow cytometry analyses were performed on synovial cells from mice with local septic arthritis pretreated with anti-RANKL antibodies. The results showed no significant differences in the frequency of infiltrating neutrophils (Supplemental Figure 4, A–C) or monocyte subsets (Supplemental Figure 4, D–F) between mice treated with anti-RANKL antibodies and those treated with control antibodies. Additionally, anti-RANKL treatment did not alter the expression of cellular markers, including RANK, c-Fms, CCR2, CD64, and CX3CR1, on infiltrating monocytes (Supplemental Figure 4, G–M).

### Combination therapy with anti-RANKL treatment and cloxacillin is superior to cloxacillin alone in protecting against joint damage in septic arthritis.

**Figure 6:**
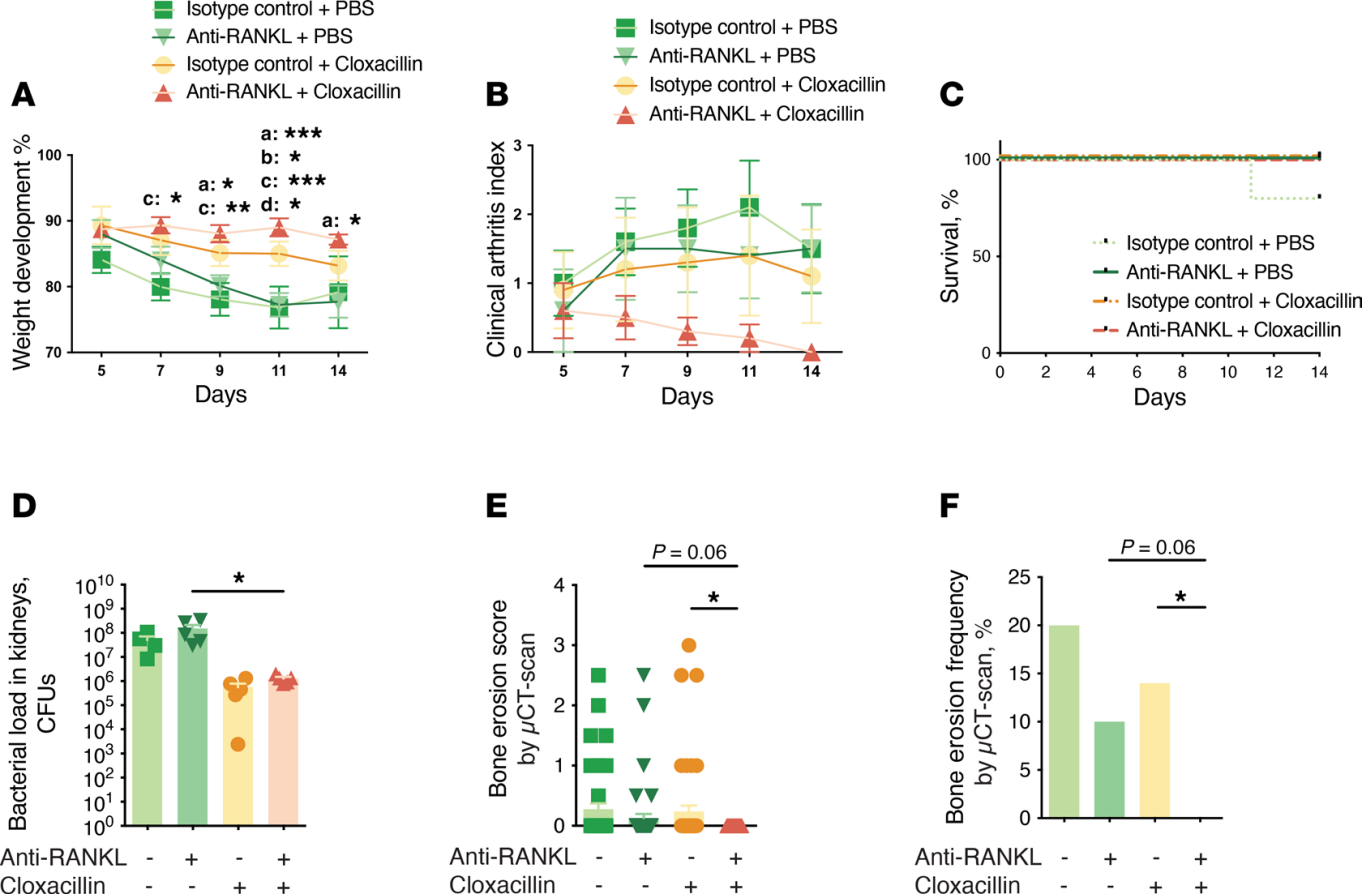
Combining antibiotics with anti-RANKL treatment proves superior to antibiotics alone in preventing joint damage in mice with septic arthritis. NMRI mice (*n* = 5/group) were intravenously injected with *S*. *aureus* Newman strain (5 × 10^6^ CFU/mouse) and sacrificed on day 14 after infection. The following treatments were administered intraperitoneally twice daily commencing on day 5 after infection to the respective groups: isotype antibodies with PBS, anti-RANKL antibodies with PBS, isotype antibodies with cloxacillin, and anti-RANKL antibodies with cloxacillin. (**A**) The changes in body weight, (**B**) arthritis severities, (**C**) cumulative survival, (**D**) bacterial loads in the kidneys, (**E**) cumulative bone destruction scores, and (**F**) frequencies of bone destruction of the joints from all 4 limbs were assessed by μCT scan. Data are presented as mean with SEM. Statistical evaluations were performed using 2-way ANOVA with Tukey’s multiple-comparison test (**A** and **B**), log-rank (Mantel-Cox) test (**C**), 1-way ANOVA with Holm-Šídák multiple-comparison test (**D**), Kruskal-Wallis test with Dunn’s multiple-comparison test (**E**), or Fisher’s exact test (**F**). a, anti-RANKL + PBS vs. anti-RANKL + cloxacillin; b, anti-RANKL + PBS vs. isotype control + cloxacillin; c, anti-RANKL + cloxacillin vs. isotype control + PBS; d, isotype control + cloxacillin vs. isotype control + PBS. *P < 0.05; **P < 0.01; ***P < 0.001.

To mimic closely the clinical scenario, young mice (6–12 weeks old) were further treated with a combination of cloxacillin and anti-RANKL antibody (or each separately) on day 5 after infection. All the mice that received cloxacillin exhibited improved body weight development, irrespective of whether they also received anti-RANKL treatment. The anti-RANKL treatment group showed body weight development similar to that of the PBS control group (Figure 6A). There was no significant difference observed between the groups regarding the clinical arthritis index (Figure 6B). Only 1 mouse in the control antibody-treated group succumbed during the experiment, while all mice in other treatment groups survived (Figure 6C). In line with the body weight development data, cloxacillin treatment effectively reduced the bacterial loads in the kidneys, whereas anti-RANKL treatment alone had no impact on bacterial proliferation in the kidneys (Figure 6D). Remarkably, the combination therapy resulted in the lowest bone erosion scores and frequencies among the treatment groups (Figure 6, E and F).

To broaden the scope of our findings, we evaluated the effects of combination therapy versus antibiotics alone in skeletally mature animals, including both young adult and middle-aged mice. Consistent with data from young mice, no significant differences were found between groups in terms of the severity of clinical arthritis, mortality, and bacterial load in kidneys of young adult (Supplemental Figure 5, A–C) and middle-aged mice (Supplemental Figure 5, F–H). In the young adult experiment, a numerically higher number of mice succumbed in the combination therapy group (Supplemental Figure 5B), whereas in the middle-aged mice, all animals in both treatment groups survived (Supplemental Figure 5G). Importantly, our data demonstrate that the combination therapy with antibiotics and anti-RANKL is more effective than antibiotics alone in preventing joint damage, both in young adult (Supplemental Figure 5, D and E) and in middle-aged mice (Supplemental Figure 5, I–K).

## Discussion

Septic arthritis poses a substantial medical challenge owing to its rapid progression and potential for irreversible joint damage. In the present study, we provide insights into the intricate mechanism underlying bone destruction in septic arthritis. Our findings demonstrate that inflammatory monocytes, characterized by high-level expression of Ly6C, play a central role in driving joint inflammation and subsequent bone resorption. Furthermore, our study highlights the critical involvement of osteoclastogenic molecules, particularly RANKL, in the process of bone destruction. Our data underscore the potential of a targeted therapeutic intervention involving anti-RANKL treatment in combination with antibiotics, to mitigate the rapid bone erosion caused by septic arthritis.

Innate immunity plays a protective role in the onset of hematogenous septic arthritis (23). Neutrophil depletion (24) or a deficiency in complement factor 3 (25) has been observed to increase the occurrence of septic arthritis in mice. This finding is attributed to reduced bacterial clearance in the bloodstream, giving the bacteria a greater opportunity to reach the joint cavity and induce the disease (26). Indeed, we also observe that neutrophils play a protective role in mitigating joint damage by eliminating the invading bacteria in a locally induced septic arthritis model. In contrast, as part of the innate immune system, monocytes paradoxically contribute to bone destruction, as our data clearly demonstrate that a lack of monocytes leads to decreased severity of joint destruction. Similar findings have been noted in a hematogenous septic arthritis model (27), reinforcing the pathogenic role of monocytes in bone destruction associated with septic arthritis. Strikingly, monocytes are also involved in osteoarthritis, as monocyte recruitment causes cartilage destruction mediated by CCL2/CCR2 in experimental osteoarthritis (28). It is noteworthy that increased mortality occurs in both the hematogenous (27) and locally induced septic arthritis models in the current study, which suggests that monocyte depletion is associated with an increased mortality rate from infections. This phenomenon appears to be associated with an elevated bacterial burden in the blood and kidneys, along with reduced systemic levels of TNF-α and IL-6, resulting in higher mortality in mice with hematogenous *S*. *aureus* septic arthritis (27). A similar mechanism, such as increased systemic bacterial dissemination, may also explain the high mortality observed in monocyte-depleted mice with locally induced septic arthritis in current study. Nevertheless, given these associated risks, broad monocyte depletion is not suitable for clinical applications. To minimize potential side effects, we identify more specific targets, rather than depleting the entire monocyte population.

Monocytes, being a heterogeneous group of antigen-presenting cells, are commonly divided into pro-inflammatory (CD11b^+^Ly6C^hi^) and antiinflammatory (CD11b^+^Ly6C^lo^) subsets (29) and further subdivided into the following subsets based on the expression of Ly6C, CCR2, and CX3CR1: Ly6C^hi^CCR2^hi^CX3CR1^int^ (pro-inflammatory) and Ly6C^lo^CX3CR1^hi^CCR2^–^ (antiinflammatory) (30). Our study reveals greater numbers of pro-inflammatory monocytes that are expressing high levels of Ly6C in the synovium at 3 days after infection. Osteoclasts, which are the only cells responsible for bone degradation (31), arise not only from within the bone but also from mature cells of the monocytic lineage in favorable microenvironments (18). Given the pivotal role of monocytes in controlling bone erosion in septic arthritis, we hypothesize that the transformation of monocytes into bone-destructive osteoclast-like cells is central to joint damage. We have analyzed the expression levels of key molecules on monocytes that are implicated in this process, such as CCR2, RANK, and c-Fms. CCR2 serves as the receptor for CCL2 (MCP-1), which is a primary chemokine for monocyte migration and activation (32, 33). Meanwhile, RANK and c-Fms are crucial receptors for osteoclastogenesis and osteoclast differentiation (34, 35). Furthermore, the expression levels of RANK, c-Fms, and CCR2 strongly correlate with Ly6C expression. We propose that these pro-inflammatory monocytes undergo transformation into osteoclast-like cells, promoting bone damage in the presence of a favorable microenvironment.

Notably, the arthritogenic and bone-destructive potentials of antibiotic-killed *S*. *aureus* explain why many patients with septic arthritis experience persistent joint inflammation and destruction despite effective antibiotic therapy. This is underscored by the standard treatment protocol of joint drainage to remove pus in septic arthritis. Among the different bacterial components, *S*. *aureus* Lpp has been identified as the most potent arthritogenic stimulus in a murine model (36). Here, we provide experimental evidence for the presence of a favorable microenvironment for bone-destructive osteoclast-like cells within the joints. In the current study, *S*. *aureus* Lpps, particularly the lipid moiety, induce the production of CCL2, MCSF, and RANKL in Lpp-injected joints. Di-acylated lipopeptides exhibit greater potency in inducing the expression of these molecules than tri-acylated lipopeptides. This is consistent with previous reports indicating that di-acylated lipopeptides are more effective than tri-acylated lipopeptides at inducing pro-inflammatory cytokines (20). Our observation that monocyte depletion markedly decreases RANKL levels while leaving the levels of MCP-1 and MCSF unaffected suggests that monocytes not only express RANK but also influence RANKL expression in other cells. It has been shown previously that during inflammatory arthritis, the formation of osteoclasts and bone erosions are primarily driven by RANKL expression on synovial fibroblasts, rather than on T cells (37). Indeed, our data demonstrate that *S*. *aureus* Lpps stimulate primary mouse synovial fibroblasts to express RANKL and that the lipid moiety of lipoproteins is responsible for this effect.

In murine models, notable bone erosion in the septic arthritis joints becomes evident 5–7 days after disease onset (11). This rapid bone destruction can lead to secondary osteoarthritis. Patients with a history of septic arthritis experience accelerated development of osteoarthritis. Specifically, knee arthroplasty rates for persons in the age range of 40–49 years are 27 times higher than for the general population of the same age (6). While our experimental models provide valuable insights into the pathogenesis of septic arthritis, they may not fully recapitulate the complexity of the disease in humans. Further randomized clinical trials are warranted to explore the long-term effects and potential adverse events associated with anti-RANKL treatment in clinical settings. Denosumab (Prolia), which is a monoclonal antibody targeting RANKL, has been approved since 2010 for the treatment of severe osteoporosis (38), showing efficacy in terms of reducing fractures and increasing bone mineral density (39, 40). Denosumab has a favorable safety profile, despite rare occurrences of osteonecrosis of the jaw and atypical femur fractures (41). This opens the door for future clinical trials that explore the combination of denosumab and conventional therapy in patients with septic arthritis. While our experimental settings show neither exacerbation of infection severity nor immunosuppression being associated with anti-RANKL treatment, it is important to note that denosumab therapy has been associated with a higher risk of infection during the early stages of treatment (42). In the current study, there were numerically more deaths observed in the anti-RANKL–treated groups in some experiments, though no statistically significant differences were detected (Supplemental Figure 6). This suggests that a cautious approach should be taken in future clinical trials and that anti-RANKL treatment might be most appropriate after bacterial culture results are obtained.

Septic arthritis is known to cause rapid systemic bone resorption with decreased bone mineral density (43). Recent studies have demonstrated that this systemic bone resorption effect caused by septic arthritis may be driven by *S*. *aureus* Lpps, with their lipid moiety specifically activating monocytes and macrophages (44). Elderly individuals are particularly vulnerable to severe osteoporosis in the context of septic arthritis. This heightened risk is due to advanced age being a major factor for septic arthritis and the high prevalence of osteoporosis in this population, with the joints affected by infection being especially susceptible. Therefore, anti-RANKL therapy may offer additional benefits by addressing osteoporosis-related complications while simultaneously enhancing joint health in these patients.

**Figure 7:**
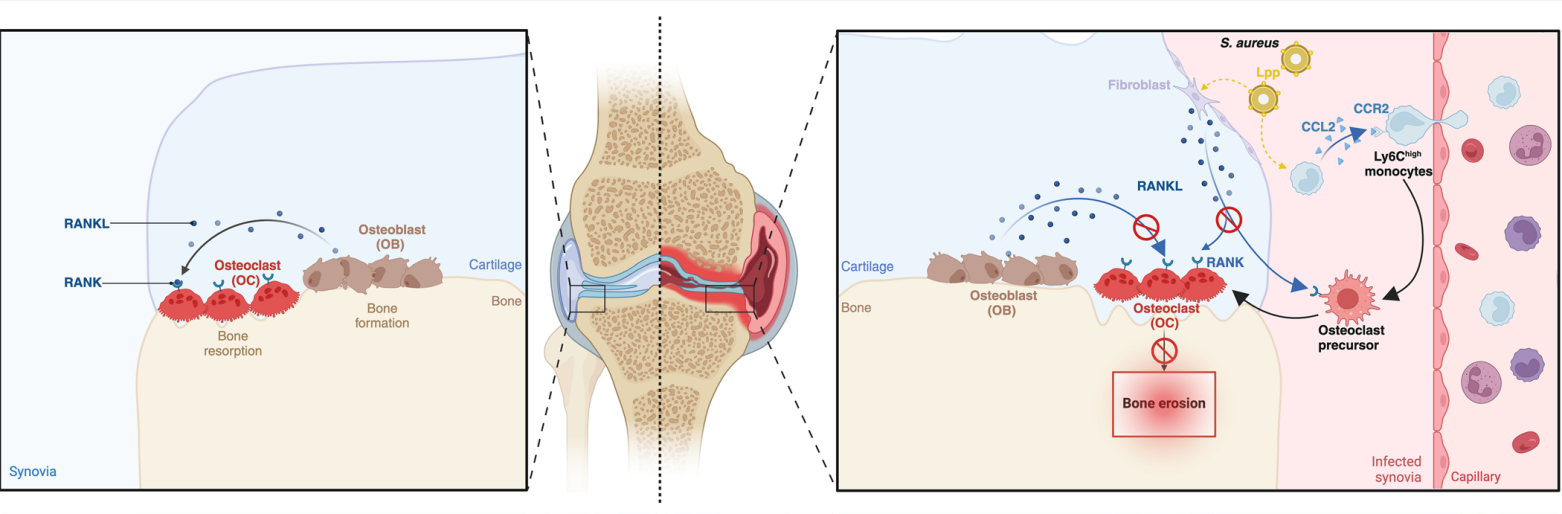
Modulating the monocyte transition to osteoclast-like cells to prevent bone destruction in septic arthritis. Schematic delineating the key steps in preventing bone destruction during septic arthritis. In the complex cascade that leads to bone damage, the recruitment of Ly6C^hi^ monocytes emerges as a pivotal event in the initiation of bone damage. *S*. *aureus* Lpps exert profound impacts on the upregulation of molecules associated with bone destruction. Targeting the RANKL proves to be effective in preventing bone damage in both locally induced and hematogenous septic arthritis models. The approach using anti-RANKL in combination with cloxacillin treatment synergistically shields against bone destruction, posing a potentially novel strategy to combat septic arthritis–induced bone damage.

In conclusion, our data suggest that the migration of monocytes to the local synovium and their subsequent transformation into bone-destructive osteoclasts represent the primary mechanism underlying joint damage in septic arthritis. The combination of anti-RANKL treatment and antibiotics is proven to be superior to antibiotics alone in preventing bone erosion in septic arthritis (Figure 7). These findings pave the way for the development of targeted therapies aimed at protecting against joint damage, preserving joint function, and improving clinical outcomes in patients with septic arthritis.

## Methods

### Sex as a biological variable.

Septic arthritis is not a sex-specific condition. A recent study comparing male and female mice found no marked differences in disease progression (45). The choice to use female mice in this study was based purely on practical considerations, as they are generally easier to handle. However, the findings may be applicable to both sexes, though further studies would be needed to confirm potential sex-specific differences.

### Mice.

Female NMRI mice, aged 6–12 weeks (young), 15 weeks (young adult), and 36 weeks (middle-aged), were purchased from Envigo. Mice were housed under standard environmental conditions of temperature and light and had free access to laboratory chow and water in the animal facility of the University of Gothenburg.

### Preparation of bacterial solutions.

The *S*. *aureus* Newman strain and LS-1 strain were prepared as described previously (46, 47).

### In vivo immune cell depletion procedures.

Depletion of both synovium-resident macrophages and systemic monocytes was achieved using clodronate liposomes (Liposoma BV) (14). For local arthritis induced by *S*. *aureus*, 200 μL of clodronate liposomes or PBS control liposomes (Liposoma) were administered intravenously (i.v.) the day before *S*. *aureus* exposure and continued on days +1, +3, and +7 after challenge. For arthritis induced by the synthetic lipopeptide Pam2CSK4 (P2C; EMC), i.a. injections of 20 μL of clodronate liposomes or PBS control liposomes were administered into the knee joints 1 day prior to P2C exposure. In addition, 200 μL of clodronate liposomes or PBS control liposomes were administered i.v. the day before P2C exposure and continued again on day +1.

Selective depletion of murine blood neutrophils was accomplished using the anti-Ly6G monoclonal antibody (clone 1A8; BioXCell), as previously documented (48). NMRI mice received intraperitoneal (i.p.) injections of 400 μg of anti-Ly6G or isotype control (clone 2A3; BioXCell) in 200 μL of PBS per mouse. These injections were administered 1 day before and on days +1 and +4 after challenge. The efficacy of cell depletion was verified by flow cytometry (Supplemental Figure 1).

### Anti-RANKL and anti-MCSFR treatments.

Monoclonal anti-RANKL antibody (clone IK22/5; BioXCell) (49) and anti-mouse CSF1R antibody (clone AFS98; BioXCell) (50) were utilized to inhibit murine RANKL and MCSFR (also known as CSF1R), respectively. NMRI mice received i.p. injections of 250 μg of either anti-RANKL or anti-CSF1R, or an isotype control antibody (clone 2A3; BioXCell), in 200 μL of PBS per mouse. For pretreatment infection experiments, the anti-RANKL injections were administered the day before the challenge. To block MCSFR activity, the anti-CSF1R treatment was given 3 hours prior to infection. Thereafter, both treatments were given 3 times per week until the end of the experiment.

### Expression and purification of Lpl1(+sp) and Lpl1(-sp).

The *S*. *aureus* Lpps Lpl1(+sp) and Lpl1(-sp) were purified and offered by Minh-Thu Nguyen (Institute of Medical Microbiology, University Hospital Münster, Münster, Germany), as previously described (51). Briefly, Lpl1(+sp) was extracted from the membrane fraction of *S*. *aureus* SA113 (pTX30::*lpl*1-his), while Lpl1(-sp) was obtained from the cytoplasmic fraction of *S*. *aureus* SA113*Δlgt* [pTX30::*lpl*1(-sp)-his] strains. Protein expression was induced using xylose (Brackett-Oliphant medium, a standard culture medium), and bacterial cells were lysed with lysostaphin (MilliporeSigma). Cytoplasmic and membrane fractions were separated by ultracentrifugation at 235,000*g* for 45 minutes at 4°C, followed by Ni-NTA affinity chromatography for purification. The proteins were washed, eluted with imidazole, concentrated, dialyzed, and lyophilized. Purity and quantity were assessed via SDS-PAGE. The final products were stored at –70°C and prepared in PBS before experiments.

### Experimental protocols for the induction of various arthritides.

To investigate the arthritogenic potentials of host immune cells and *S*. *aureus*, 5 distinct sets of experiments were performed. Mice were i.a. injected in the knee joint with 20 μL of PBS that contained one of the following: (a) live *S*. *aureus* LS-1 strain (4 × 10^3^ CFU/knee); (b) purified *S*. *aureus* Lpps (4 μg/knee), i.e., either Lpl1(+sp) or Lpl1(-sp); (c) synthetic lipopeptides (4 μg/knee), i.e., P2C or Pam3CSK4 (P3C) (EMC); (d) P2C (4 μg/knee), given subsequent to monocyte depletion; and (e) live *S*. *aureus* LS-1 strain (4 × 10^3^ CFU/knee), injected after blocking MCSF or RANKL. Clinical severity was evaluated by measuring the differences in knee joint diameters with a caliper.

### Experimental protocol for the S. aureus septic arthritis mouse model.

To study the effect of anti-RANKL treatment on septic arthritis, our well-established hematogenous septic arthritis model was applied (52). In brief, a 200 μL suspension of *S*. *aureus* Newman strain (5 × 10^6^ CFU/mouse) was inoculated into the tail veins of the mice. The mice were monitored by 3 observers who were blinded to the treatment groups, on a daily basis until experimental termination, i.e., up to 10 or 14 days after infection. The severity of arthritis was assessed using a clinical scoring scale, ranging from 0 to 3 (52). On the termination day, blood, kidneys, and paws were collected.

### Bacteriologic examinations of kidneys and joints.

Kidney and joint samples were homogenized, serially diluted, and plated on horse blood agar plates for the quantification of bacterial CFU, as described previously (53).

### Flow cytometry.

Knee synovial tissue was collected and placed in a tube that contained RPMI medium (Thermo Fisher Scientific). After adding type IV collagenase (Thermo Fisher Scientific) and DNase I (MilliporeSigma) according to the manufacturers’ instructions, the tissue was incubated for 1 hour at 37°C. Following this, a single-cell suspension was obtained through tissue homogenization and filtration using a 40 μm cell strainer (Falcon, Corning). Synovial cells were processed for flow cytometric analysis following established procedures (14). The cells were blocked with Mouse BD Fc Block (BD Biosciences) and labeled with an antibody cocktail (Supplemental Table 1). UltraComp eBeads Compensation Beads (Invitrogen) were used for the compensation setup. Fluorescence minus 1 samples were used to identify the negative population for each antibody. Sample acquisition was performed using CytoFLEX S (Beckman Coulter), and the data were analyzed using the FlowJo software (v. 10.10; Tree Star). Representative images of the gating strategy are shown in Supplemental Figure 2.

### μCT.

All 4 paws from the mice were scanned using a SkyScan 1176 micro-CT (Bruker). The NRecon software (v. 1.6.9.8; Bruker) was used to reconstruct 3-dimensional images, and the CTvox software (v. 2.7.0; Bruker) was subsequently used for the evaluation. Each joint was evaluated using a scoring system as previously described (11).

### Histopathologic examination and immunohistochemistry.

After μCT scanning, the joints were subjected to decalcification, followed by paraffin embedding and microtome sectioning. The resulting tissue sections were stained with TRAP. The staining was performed using a customized TRAP buffer that consisted of 0.2 M acetate buffer, 0.3 M sodium tartrate, 10 mg/mL naphtol AS-MX phosphate, 0.1% Triton X-100, and 0.3 mg/mL Fast Red Violet LB (MilliporeSigma). Following deparaffinization and incubation with acetate buffer, the tissue samples were incubated in the TRAP buffer overnight and subsequently counterstained with Fast Green (54).

Immunohistochemistry was conducted using the MACH1 Universal HRP-Polymer Detection Kit (Biocare Medical). All procedures were performed at room temperature unless otherwise specified, as described previously (55). Briefly, after deparaffinization and rehydration, sections underwent antigen retrieval in Tris/EDTA buffer (pH 9) using microwave heating at medium power for 2 rounds of 5 minutes. Endogenous peroxidase was blocked for 5 minutes using Peroxidazed 1 (Biocare Medical). Nonspecific protein binding was blocked with Background Sniper (Biocare Medical) for 15 minutes. The slides were then incubated overnight at 4°C in a moist chamber with primary antibody: rabbit monoclonal anti-CD68 (EPR23917-164; Abcam), diluted 1:2,000 in Da Vinci Green Diluent (Biocare Medical). After overnight incubation, the MACH1 Universal HRP-Polymer (Biocare Medical) was added, and the slides were incubated in a moist chamber for 30 minutes. Staining was carried out using DAB solution (Biocare Medical). The sections were then counterstained with Mayer’s hematoxylin (Histolab Products AB). After drying in an oven at 60°C for 15–20 minutes, the sections were mounted with Pertex (Histolab) and photographed under a light microscope. Positive immunoreactivity was indicated by brown staining. Negative controls were conducted by excluding the primary antibody and incubating the tissues with Da Vinci Green Diluent instead, resulting in no immunoreactivity.

### Measurement of cytokine/chemokine levels in knee homogenates.

Knee joints, with the surrounding tissues removed, were collected and homogenized as previously described (19). The levels of RANKL, MCP-1 (CCL2), and MCSF in the knee homogenates were quantified using DuoSet ELISA Kits (R&D Systems Europe) according to the manufacturer’s instructions.

### RANKL gene expression in mouse synovial fibroblasts.

Primary murine fibroblasts were cultured in fibroblast growth media that contained Dulbecco’s modified Eagle medium (DMEM) (Gibco), 10% fetal calf serum (FCS) (Gibco), 50 μg/mL gentamicin (Gibco), 1,000 U/mL penicillin, and 1,000 μg/mL streptomycin (Gibco), at 37°C and 5% CO_2_. Fibroblasts were seeded in 6-well plates in fibroblast growth media. Once they reached confluence, the fibroblasts were starved for 24 hours in fibroblast starvation media (DMEM, containing 0.1% FCS, 50 μg/mL gentamicin, 1,000 U/mL penicillin, and 1,000 μg/mL streptomycin) before being stimulated with P2C or P3C or with Lpl1(+sp) or Lpl1(-sp). After 48 hours, the cells were suspended in 1× QIAzol (QIAGEN), and mRNA extraction was performed with the miRNeasy Mini Kit (QIAGEN), following the manufacturer’s protocol. cDNA synthesis was carried out using the SuperScript III kit (Invitrogen) according to the manufacturer’s instructions.

The expression levels of *RANKL* (*Tnfsf11*, Mm00441906_m1) were analyzed with the TaqMan Universal PCR Master Mix kit (Applied Biosystems), and *Actb* (Mm00607939_s1) was used as an internal control. The analysis was conducted with the ViiA 7 Fast Real-Time PCR system (Applied Biosystems). All the samples were run in duplicate, and the relative expression values were calculated using the ΔCt or ΔΔCt method, as indicated.

### Statistics.

Statistical analyses were performed using the GraphPad Prism 9 software. Comparisons among experimental groups were assessed using the Mann-Whitney *U* test, Fisher’s exact test, 1- or 2-way ANOVA test, or Kruskal-Wallis test, as appropriate. Survival was assessed with a log-rank (Mantel-Cox) test. All results are reported as the median or mean ± SEM unless indicated otherwise. A *P* < 0.05 was considered statistically significant.

### Study approval.

Mouse studies were reviewed and approved by the Ethics Committee of Animal Research of Gothenburg and conducted in accordance with the regulations and recommendations of the Swedish Board of Agriculture for animal experiments.

### Data availability.

All the raw and processed data are stored at the University of Gothenburg and are available upon request. Values for all data points in graphs are reported in the Supporting Data Values file.

## Author contributions

TJ, ZH, MM, RP, CE, and MNDS conceptualized and designed the experiments. ZH conducted the experiments, with assistance from MD, AJ, MB, PKK, and AA. CC provided expertise and reagents for TRAP staining. MNDS provided mouse knee synovial fibroblasts and cell culture reagents. ZH drafted the manuscript, which was reviewed and edited by MM and TJ. ZH, MD, AJ, MB, CC, PKK, AA, MNDS, CE, RP, MM, and TJ contributed to data interpretation; provided critical feedback; and contributed to the refinement of the research, analysis, and manuscript. All authors approved the final version of the manuscript.

## Supplementary Material

Supplemental data

Supporting data values

## Version 1 03/11/2025

In-Press Preview

## Version 2 03/24/2025

Electronic publication

## References

[1] Mathews CJ (2010). Bacterial septic arthritis in adults. Lancet.

[2] Gunnlaugsdottir SL (2022). Native joint infections in Iceland 2003-2017: an increase in postarthroscopic infections. Ann Rheum Dis.

[3] Geirsson AJ (2008). Septic arthritis in Iceland 1990–2002: increasing incidence due to iatrogenic infections. Ann Rheum Dis.

[4] Kaandorp CJ (1997). The outcome of bacterial arthritis: a prospective community-based study. Arthritis Rheum.

[5] Bohle S (2024). Incidence of secondary osteoarthritis after primary shoulder and knee empyema and its risk factors. J Pers Med.

[6] Abram SGF (2020). Mortality and adverse joint outcomes following septic arthritis of the native knee: a longitudinal cohort study of patients receiving arthroscopic washout. Lancet Infect Dis.

[7] Sakiniene E (1996). Addition of corticosteroids to antibiotic treatment ameliorates the course of experimental Staphylococcus aureus arthritis. Arthritis Rheum.

[8] Fei Y (2011). The combination of a tumor necrosis factor inhibitor and antibiotic alleviates staphylococcal arthritis and sepsis in mice. J Infect Dis.

[9] Ali A (2015). CTLA4 immunoglobulin but not anti-tumor necrosis factor therapy promotes Staphylococcal septic arthritis in mice. J Infect Dis.

[10] Schett G, Gravallese E (2012). Bone erosion in rheumatoid arthritis: mechanisms, diagnosis and treatment. Nat Rev Rheumatol.

[11] Fatima F (2017). Radiological features of experimental staphylococcal septic arthritis by micro computed tomography scan. PLoS One.

[12] Deng GM (1999). Intra-articularly localized bacterial DNA containing CpG motifs induces arthritis. Nat Med.

[13] Ali A (2015). Antibiotic-killed Staphylococcus aureus induces destructive arthritis in mice. Arthritis Rheumatol.

[14] Mohammad M (2019). The YIN and YANG of lipoproteins in developing and preventing infectious arthritis by Staphylococcus aureus. PLoS Pathog.

[15] Mohammad M (2022). Staphylococcus aureus lipoproteins in infectious diseases. Front Microbiol.

[16] Walsh NC, Gravallese EM (2010). Bone remodeling in rheumatic disease: a question of balance. Immunol Rev.

[17] Takeuchi T (2019). Effects of the anti-RANKL antibody denosumab on joint structural damage in patients with rheumatoid arthritis treated with conventional synthetic disease-modifying antirheumatic drugs (DESIRABLE study): a randomised, double-blind, placebo-controlled phase 3 trial. Ann Rheum Dis.

[18] Udagawa N (1990). Origin of osteoclasts: mature monocytes and macrophages are capable of differentiating into osteoclasts under a suitable microenvironment prepared by bone marrow-derived stromal cells. Proc Natl Acad Sci U S A.

[19] Nguyen MT (2023). Bacterial Lipoproteins Shift Cellular Metabolism to Glycolysis in Macrophages Causing Bone Erosion. Microbiol Spectr.

[20] Nguyen MT (2017). Lipid moieties on lipoproteins of commensal and non-commensal staphylococci induce differential immune responses. Nat Commun.

[21] Croft AP (2019). Distinct fibroblast subsets drive inflammation and damage in arthritis. Nature.

[22] Khan NM (2020). Comparative transcriptomic analysis identifies distinct molecular signatures and regulatory networks of chondroclasts and osteoclasts. Arthritis Res Ther.

[23] Jin T (2021). Bacteria and host interplay in Staphylococcus aureus septic arthritis and sepsis. Pathogens.

[24] Verdrengh M, Tarkowski A (1997). Role of neutrophils in experimental septicemia and septic arthritis induced by Staphylococcus aureus. Infect Immun.

[25] Na M (2016). Deficiency of the complement component 3 but not factor B aggravates Staphylococcus aureus septic arthritis in mice. Infect Immun.

[26] Jin T (2024). Exploring the role of bacterial virulence factors and host elements in septic arthritis: insights from animal models for innovative therapies. Front Microbiol.

[27] Verdrengh M, Tarkowski A (2000). Role of macrophages in Staphylococcus aureus-induced arthritis and sepsis. Arthritis Rheum.

[28] Raghu H (2017). CCL2/CCR2, but not CCL5/CCR5, mediates monocyte recruitment, inflammation and cartilage destruction in osteoarthritis. Ann Rheum Dis.

[29] Geissmann F (2010). Development of monocytes, macrophages, and dendritic cells. Science.

[30] Hoenow S (2022). The properties of proinflammatory Ly6C(hi) monocytes are differentially shaped by parasitic and bacterial liver infections. Cells.

[31] Teitelbaum SL (2000). Bone resorption by osteoclasts. Science.

[32] Park HK (2022). Analysis of CCR2 splice variant expression patterns and functional properties. Cell Biosci.

[33] Shroka TM (2023). The dual-function chemokine receptor CCR2 drives migration and chemokine scavenging through distinct mechanisms. Sci Signal.

[34] Inoue K (2023). Bone marrow Adipoq-lineage progenitors are a major cellular source of M-CSF that dominates bone marrow macrophage development, osteoclastogenesis, and bone mass. eLife.

[35] Li Y (2019). Specific RANK cytoplasmic motifs drive osteoclastogenesis. J Bone Miner Res.

[36] Mohammad M (2020). The role of Staphylococcus aureus lipoproteins in hematogenous septic arthritis. Sci Rep.

[37] Danks L (2016). RANKL expressed on synovial fibroblasts is primarily responsible for bone erosions during joint inflammation. Ann Rheum Dis.

[38] Lu J (2023). Current comprehensive understanding of denosumab (the RANKL neutralizing antibody) in the treatment of bone metastasis of malignant tumors, including pharmacological mechanism and clinical trials. Front Oncol.

[39] Zhang N (2020). Pros and Cons of Denosumab Treatment for Osteoporosis and Implication for RANKL Aptamer Therapy. Front Cell Dev Biol.

[40] Kroupova K (2023). Monoclonal antibodies for treatment of osteoporosis. Drugs Today (Barc).

[41] Black DM, Rosen CJ (2016). Clinical Practice. Postmenopausal Osteoporosis. N Engl J Med.

[42] Huang ST (2023). Denosumab treatment and infection risks in patients with osteoporosis: propensity score matching analysis of a national-wide population-based cohort study. Front Endocrinol (Lausanne).

[43] Verdrengh M (2006). Rapid systemic bone resorption during the course of Staphylococcus aureus-induced arthritis. J Infect Dis.

[44] Schultz M (2022). Lipoproteins cause bone resorption in a mouse model of Staphylococcus aureus septic arthritis. Front Microbiol.

[45] Hu Z (2023). The impact of aging and TLR2 deficiency on the clinical outcomes of Staphylococcus aureus bacteremia. J Infect Dis.

[46] Bremell T (1991). Experimental Staphylococcus aureus arthritis in mice. Infect Immun.

[47] Kwiecinski J (2013). Staphylokinase promotes the establishment of Staphylococcus aureus skin infections while decreasing disease severity. J Infect Dis.

[48] Daley JM (2008). Use of Ly6G-specific monoclonal antibody to deplete neutrophils in mice. J Leukoc Biol.

[49] Metzger TC (2013). Lineage tracing and cell ablation identify a post-Aire-expressing thymic epithelial cell population. Cell Rep.

[50] Moynihan KD (2016). Eradication of large established tumors in mice by combination immunotherapy that engages innate and adaptive immune responses. Nat Med.

[51] Nguyen MT (2015). The nuSaalpha specific lipoprotein like cluster (lpl) of S. aureus USA300 contributes to immune stimulation and invasion in human cells. PLoS Pathog.

[52] Jarneborn A (2020). Tofacitinib treatment aggravates Staphylococcus aureus septic arthritis, but attenuates sepsis and enterotoxin induced shock in mice. Sci Rep.

[53] Hu Z (2023). The impact of aging and toll-like receptor 2 deficiency on the clinical outcomes of Staphylococcus aureus bacteremia. J Infect Dis.

[54] Mediero A (2015). Direct or indirect stimulation of adenosine A2A receptors enhances bone regeneration as well as bone morphogenetic protein-2. FASEB J.

[55] Arnsrud Godtman R (2021). Constitutive expression of inducible nitric oxide synthase in healthy rat urothelium?. Scand J Urol.

