# Supplementary material for: Combination treatment with anti-RANKL and antibiotics for preventing joint destruction in septic arthritis
Source: JCI Insight. 2025 Mar 24;10(6):e184954. doi: 10.1172/jci.insight.184954 (PMC11949037; doi:10.1172/jci.insight.184954)
Supplement: Supplemental data [file jciinsight-10-184954-s229.pdf]

Supplemental Material

Combination treatment with anti-RANKL and antibiotics for preventing joint destruction in septic arthritis

Zhicheng Hu[1,2]*, Meghshree Deshmukh[1], Anders Jarneborn[1,3], Miriam Bollmann[1,4], Carmen Corciulo[5], Pradeep Kumar Kopparapu[1], Abukar Ali[1], Mattias N. D. Svensson[1,4], Cecilia Engdahl[1], Rille Pullerits[1,6], Majd Mohammad[1], Tao Jin[1,3]

*Corresponding author

[1]Department of Rheumatology and Inflammation Research, Institute of Medicine, The Sahlgrenska Academy, University of Gothenburg, Gothenburg, Sweden

[2]Center for Clinical Laboratories, the Affiliated Hospital of Guizhou Medical University, Guiyang, China

[3]Department of Rheumatology, Sahlgrenska University Hospital, Gothenburg, Sweden

[4]SciLifeLab, University of Gothenburg, Sweden

[5]Department of Pharmacology, Institute of Neuroscience and Physiology, University of Gothenburg.

[6]Department of Clinical Immunology and Transfusion Medicine, Sahlgrenska University Hospital, Gothenburg, Sweden.

Correspondence: Zhicheng Hu, MD, PhD, Department of Rheumatology and Inflammation Research, Institute of Medicine, Sahlgrenska Academy at the University of Gothenburg, Guldhedsgatan 10A, S-413 46 Gothenburg, Sweden (e-mail: zhicheng.hu@gu.se). Phone number: +46 0790128740.

Supplemental Table 1. Antibodies used in the flow cytometry panel.

	Antibody	Fluorophore	Host	Clone	Supplier
1	CD45	APC-R700	Rat	30-F11	BD Biosciences
2	CD11b	V450	Rat	M1/70	BD Biosciences
3	Ly6G	PE-Cy7	Rat	1A8	BD Biosciences
4	Ly6C	BV605	Rat	AL-21	BD Biosciences
5	CD64	PerCP e710	Mouse	X54-5/7.1	Invitrogen
6	c-Fms (CD115)	FITC	Rat	AFS98	Proteintech
7	CX3CR1	APC	Mouse	SA011F11	BioLegend
8	CCR2 (CD192)	BV650	Rat	SA203G11	BioLegend
9	RANK (CD265)	PE	Rat	R12-31	Invitrogen
10	Viability dye	eFluor 506			Invitrogen

APC, Allophycocyanin; BV, brilliant violet; FITC, fluorescein isothiocyanate; PE, phycoerythrin; PerCP, peridinin chlorophyll protein complex.

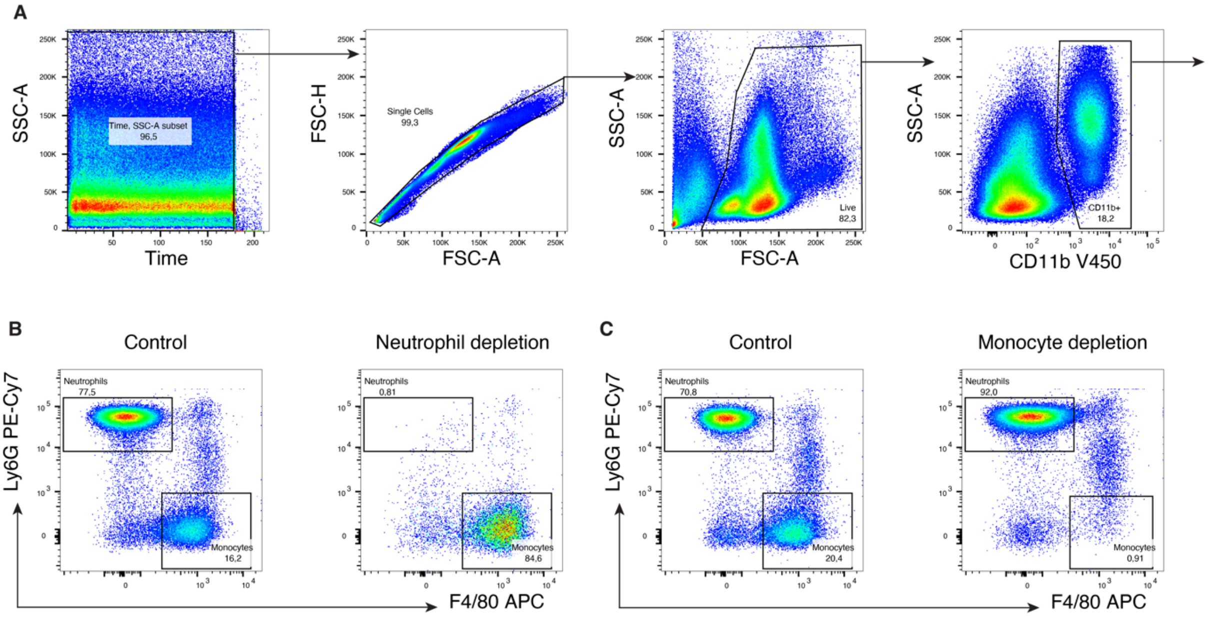

Supplemental Figure 1. Verification of monocyte or neutrophil depletion by flow cytometry. NMRI mice were subjected to treatment with anti-mouse Ly6G monoclonal antibody (mAb) to deplete neutrophils or clodronate liposomes to deplete monocytes/macrophages. Blood samples were collected 1 day after treatment. Representative images of the gating strategy (**A**), and the efficacy of cell depletion for: (**B**) neutrophils (CD11b+Ly6G+F4/80-); and (**C**) monocytes/macrophages (CD11b+Ly6G-F4/80+).

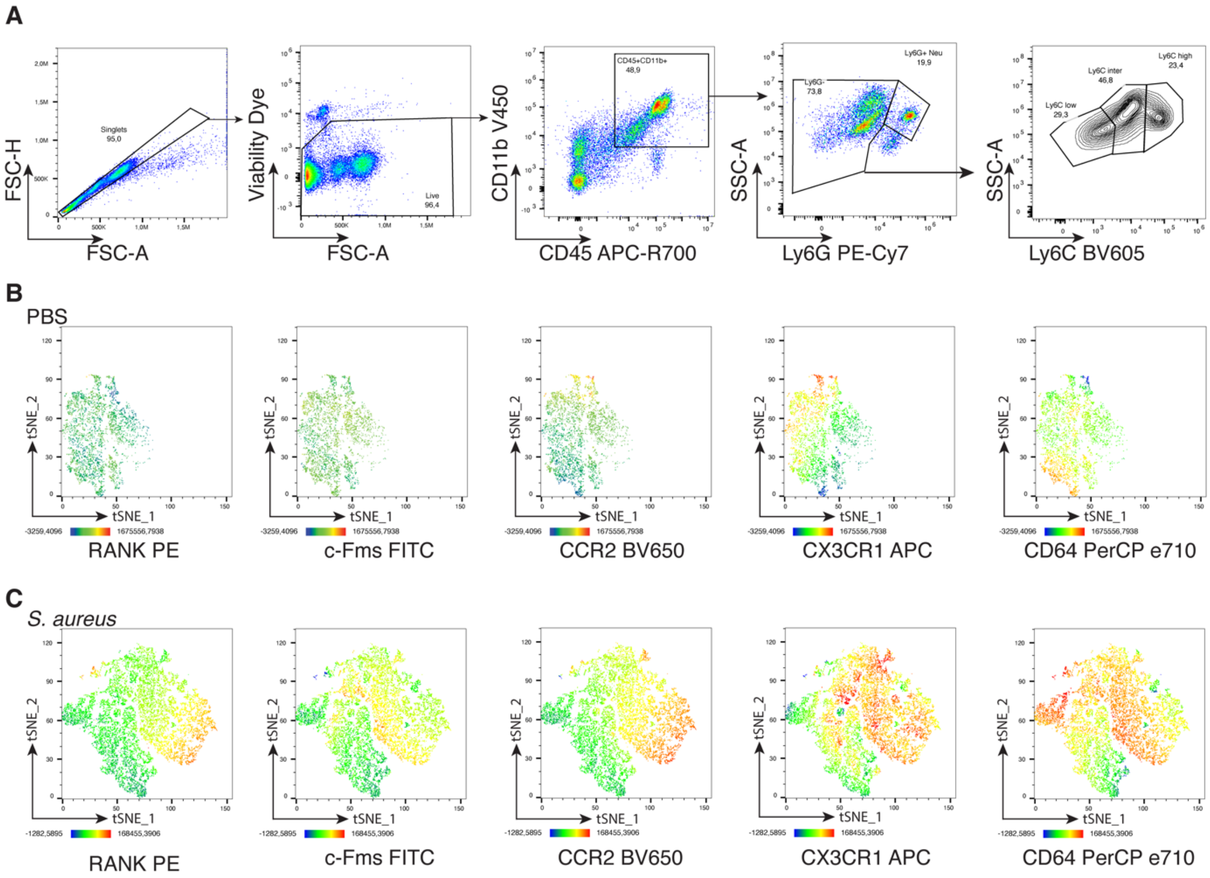

Supplemental Figure 2. Verification of dynamic changes in cellular markers for monocytes using flow cytometry. Knee synovial tissues from mice injected intra-articularly with 20 μL of PBS (n=4) or PBS containing *S. aureus* LS-1 strain (n=6) were analyzed on Day 3 post-injection using flow cytometry. (**A**) Representative images for the gating strategy. (**B** and **C**) tSNE analysis of monocyte subsets based on RANK, c-Fms, CCR2, CX3CR1 and CD64 expression levels with gating on the CD11b+CD45+Ly6G- population. Representative images are shown.

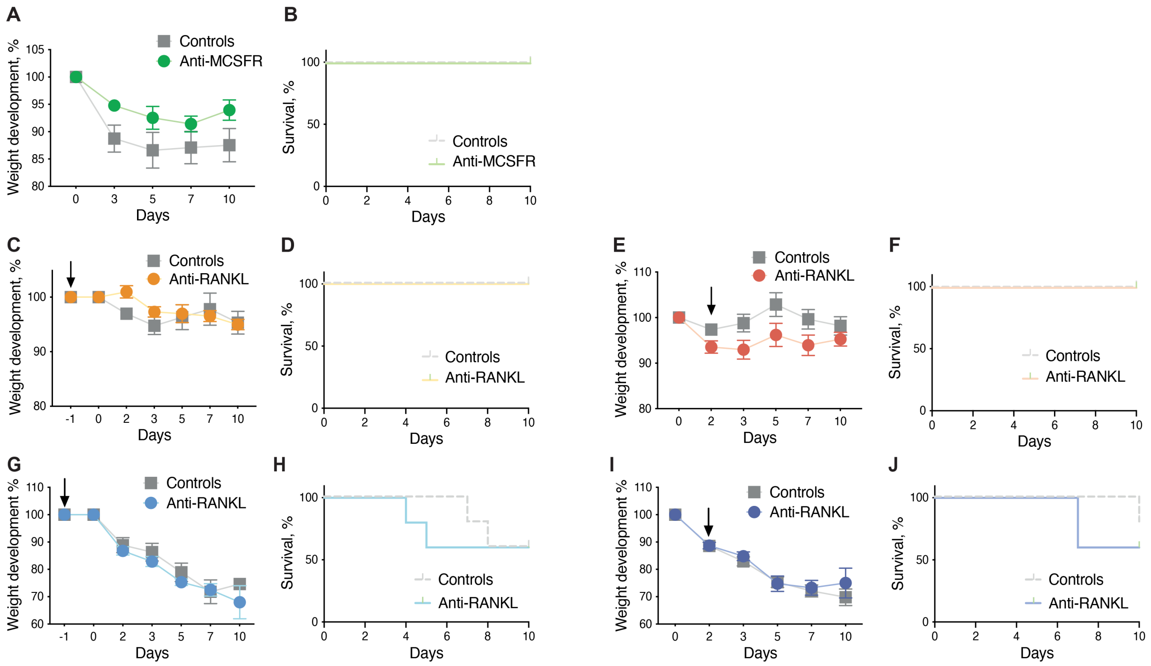

Supplemental Figure 3. Treatment with anti-RANKL or anti-MCSF antibodies has no impact on body-weight development or mortality outcomes from locally induced- and hematogenous septic arthritis models. Body-weight changes and cumulative survival were monitored for 10 days after NMRI mice (n=5/group) underwent intra-articular (i.a.) infection with 20 µL of PBS containing the *S. aureus* LS-1 strain (4 × 10^3 CFU/knee) and that received treatment with: (**A** and **B**) anti-mouse CSF1R (anti-MCSFR) antibody 3 hours prior to infection; (**C** and **D**) anti-mouse RANKL (anti-RANKL) antibody 1 day prior to infection; (**E** and **F**) anti-RANKL antibody 2 days post-infection, or isotype control antibody (Controls). In addition, to mimic hematogenic infection, NMRI mice (n = 5/group) received anti-RANKL antibody treatment or isotype antibody control 1 day before they were intravenously injected with the *S. aureus* Newman strain (5 × 10^6 CFU/mouse), and were sacrificed on Day 10 post-infection. (**G**) Body-weight changes and (**H**) mortality were monitored. (**I** and **J**) Further assessments with administration of treatment at 2 days post-infection were conducted to simulate clinical progression. The arrows indicate the start of treatments. Statistical evaluations were performed using two-way ANOVA with Sidak's multiple comparison test, with data presented as mean with SEM (A-D, and F) or log-rank (Mantel-Cox) test (E and G).

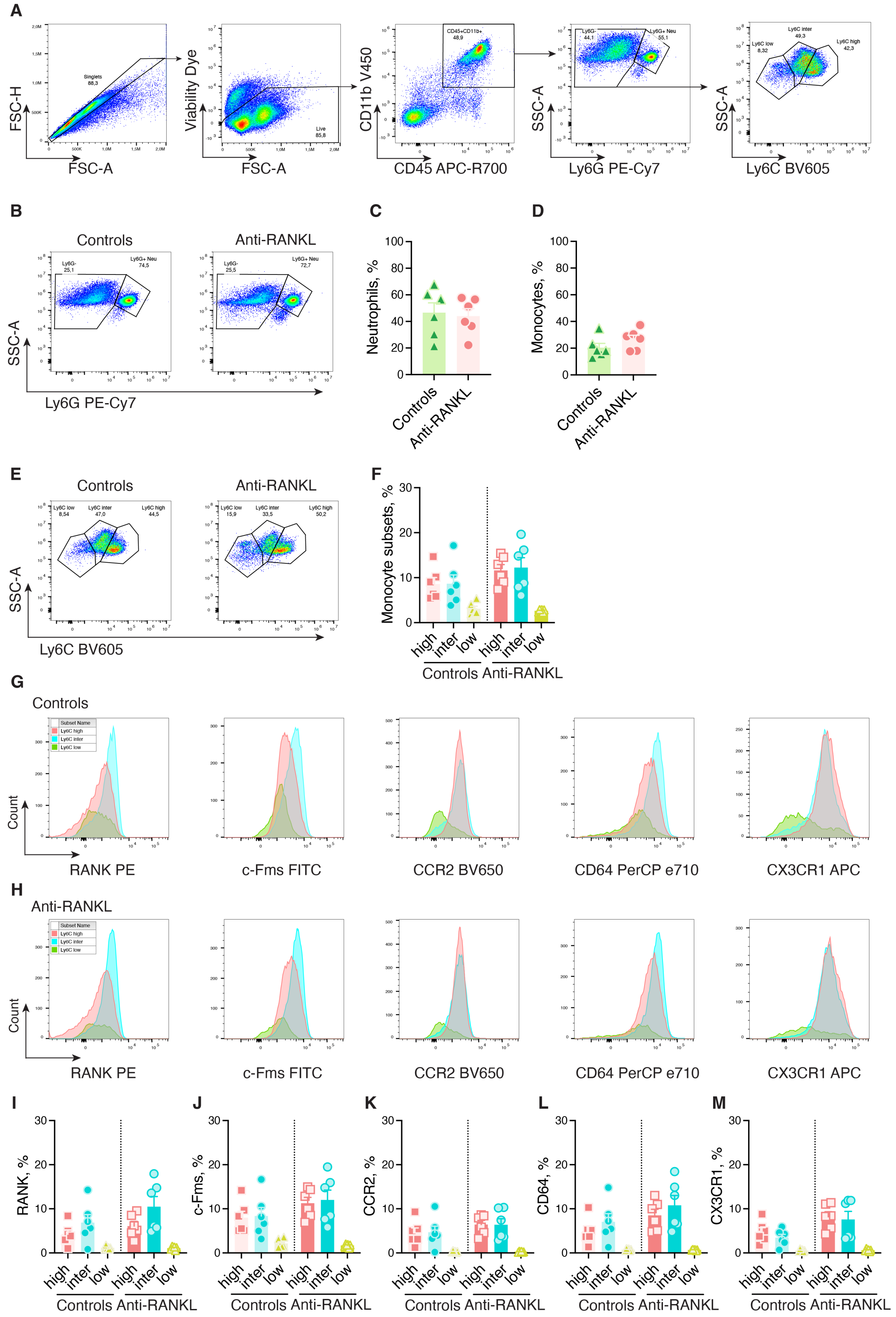

Supplemental Figure 4. Anti-RANKL treatment does not affect the infiltration of monocyte/neutrophils or the expression of cellular markers on infiltrating monocytes in knee synovial tissue following septic arthritis. NMRI mice were administered with anti-mouse RANKL antibodies (anti-RANKL) or an isotype control (Controls) 1 day prior to intra-

articular injection of 20 µL of PBS containing *S. aureus* LS-1 strain (4 × 10^3 CFU/ knee). Knee synovial tissues (n=6/group) were collected on day 3 post-injection for flow cytometry analysis. (**A**) Representative images of the gating strategy. (**B**) Gating strategy within CD11b+CD45+ population. (**C**) Neutrophils gated as CD11b+CD45+Ly6G+ cells. (**D**) Monocytes gated as CD11b+CD45+Ly6G- cells. (**E**) Gating strategy for Ly6C expression within CD11b+CD45+Ly6G+ cells. (**F**) Monocyte subsets classified based on Ly6C expression levels. Histograms showing expression levels of RANK, c-Fms, CCR2, CD64 and CX3CR1 in concatenated data sets for (**G**) Controls and (**H**) anti-RANKL groups. Proportions of monocyte subsets expressing (**I**) RANK, (**J**) c-Fms, (**K**) CCR2, (**L**) CD64 and (**M**) CX3CR1. Data are reported as mean ± SEM and analyzed with the Mann-Whitney test.

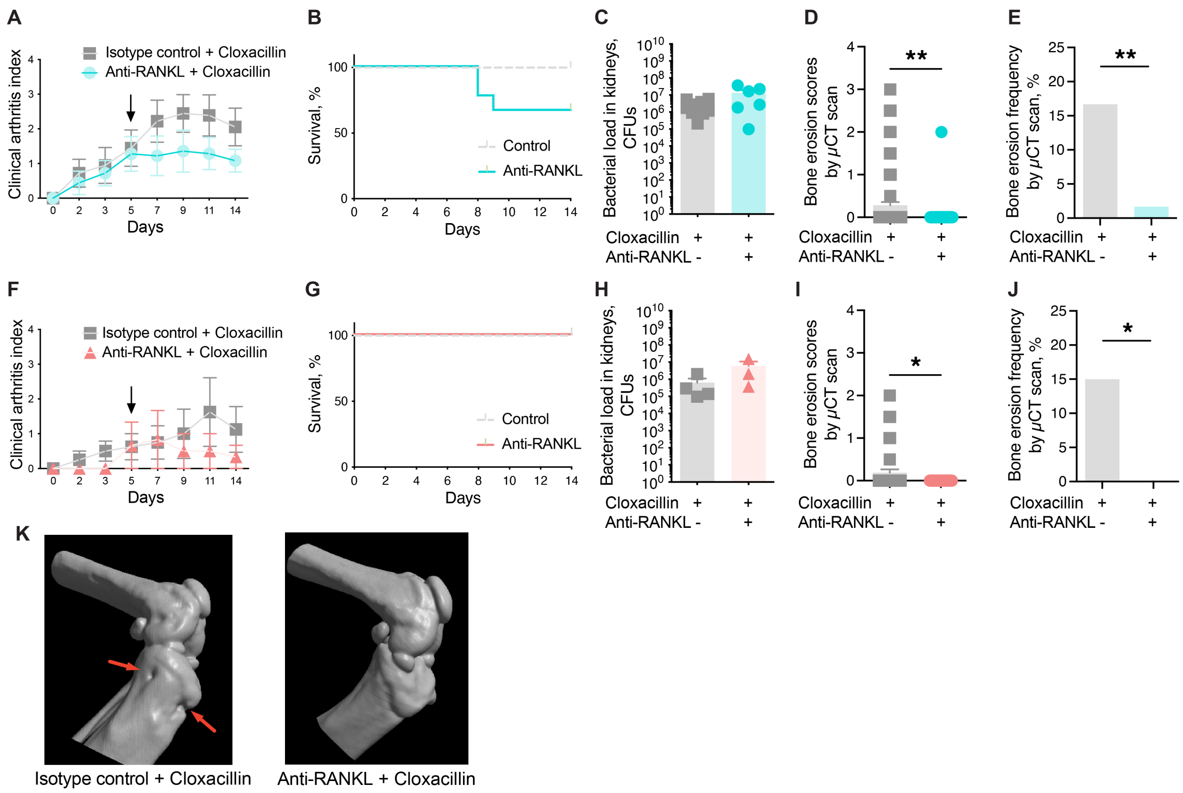

Supplemental Figure 5. Combined antibiotics and anti-RANKL treatment is superior to antibiotics alone in preventing joint damage in skeletally mature mice with septic arthritis. NMRI mice (15 weeks old) were intravenously injected with *S. aureus* Newman strain (5×10^6 CFU/mouse) and sacrificed on Day 14 post-infection. On Day 5 post-infection, mice were divided into two groups (n = 9/group) and treated with anti-mouse RANKL antibodies (anti-RANKL) or an isotype control three times per week, alongside subcutaneous cloxacillin administered twice daily. Evaluations included (**A**) changes of arthritis severities scores, (**B**) cumulative survival, (**C**) bacterial loads in kidneys, (**D**) bone destruction scores, and (**E**) frequencies of bone destruction in all four limbs joints were assessed by µCT scan. (**F-J**) The same experimental setup was repeated in middle-aged mice (36 weeks old), with treatment groups consisting of combination therapy (n=3) or antibiotics alone (n=4). (**K**) Representative µCT images of knee joints from the combination therapy group and antibiotics alone group. Arrows indicate bone erosion. Data are presented as mean ± SEM and analyzed using the Mann-Whitney test (A, C, D, F, H and I), log-rank (Mantel-Cox) test (B and G), or Fisher's exact test (E and J). **P* < 0.05; ***P* < 0.01.

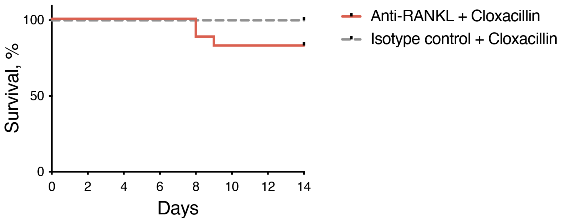

Supplemental Figure 6. Survival outcomes in mice treated with combined antibiotics and anti-RANKL therapy compared to antibiotics alone in septic arthritis. NMRI mice (6-12 weeks, 15 weeks, and 36 weeks old) were intravenously injected with *S. aureus* Newman strain (5×10^6 CFU/mouse) and sacrificed on Day 14 post-infection. On Day 5 post-infection, mice were divided into two groups and treated with anti-mouse RANKL antibodies (anti-RANKL, n=17) or isotype control (n=18) three times per week, alongside subcutaneous cloxacillin administered twice daily. Cumulative survival rates were evaluated and pooled from three independent experiments. Data were analyzed using the log-rank (Mantel-Cox) test.
